# Cloning and Function Analysis of the CsTAU1 in Response to Salt–Alkali Stress

**DOI:** 10.3390/genes15050613

**Published:** 2024-05-11

**Authors:** Fan Zhang, Dandan Li, Rina Sa, Ling Wang, Yunyan Sheng

**Affiliations:** Horticulture and Landscape Department, Heilongjiang Bayi Agriculture University, Daqing 163000, China; zhangfan2434@byau.edu.cn (F.Z.); lidandan002@byau.edu.cn (D.L.); sarina@byau.edu.cn (R.S.); wangling@byau.edu.cn (L.W.)

**Keywords:** cucumber, *CsTAU1*, salt–alkali stress, functional analysis

## Abstract

To investigate the role of candidate genes for salt–alkali tolerance in cucumber (*Cucumis sativus* L.), this study screened *CsTAU1* in the glutathione pathway from previous transcriptome data for cloning and functional analysis. Clone cucumber *CsTAU1* contains one 675 bp open reading frame, containing one GST-N-Tau domain and one GST-C-Tau domain, and is expressed in cytoplasm. After successfully constructing overexpression vectors of CsTAU1 (+) and CsTAU1 (−), they were transferred into cucumber varieties ‘D1909’ (high salt alkali resistance) and ‘D1604’ (low salt alkali resistance) for salt–alkali resistance identification. It was found that under salt–alkali stress, CsTAU1 (+)-overexpressing plants showed strong resistance to salt–alkali stress, while CsTAU1 (−)-overexpressing plants showed the opposite situation. qRT-PCR analysis was performed on other glutathione pathway-related genes in *CsTAU1*-overexpressing plants. The expression patterns of LOC101219529 and LOC105434443 were the same as *CsTAU1*, and the introduction of *CsTAU1* (+) increased the chlorophyll, α-Naphthylamine oxidation, glutathione S-transferase (GST), and catalase (CAT) content of cucumber. The research results provide a theoretical basis for cultivating salt–alkali-tolerant cucumber varieties.

## 1. Introduction

In recent years, cucumber has gradually become one of the most popular fruits and vegetables in the market, and is widely produced around the world. Daqing is located in the Songnen Plain of China, and it has sulfate soda saline–alkali soil [1]. Saline–alkali soil is not conducive to the growth of cucumbers. Even if it can grow, its economic value will be affected because salt–alkali stress can lead to poor root development, weakened absorption capacity, and destruction of organic matter accumulation in cucumbers. Additionally, saline–alkali soil can also pollute groundwater, affecting human production and domestic water use [2]. Breeding salt–alkali-tolerant germplasm resources can effectively promote agricultural income and efficiency for farmers, and form a new layout of green industry [3]. Research on cucumber saline–alkali tolerance has gradually become a hot topic. As one of the three major regions with concentrated distribution of saline soil in the world, Daqing should pay more attention to the research on saline–alkali tolerance [4].

Glutathione (GSH) is one of the antioxidant substances widely present in plants, playing a role in antioxidant defense, cell detoxification, free radical neutralization, and other aspects, and it is an important enzyme activity regulatory substance [5]. GSH can participate in biological transformation, thereby converting harmful substances produced after salt–alkali stress into harmless substances and excreting them from the plant [6]. It can also directly induce the expression of genes related to salt–alkali tolerance in cucumber, thereby activating the antioxidant substances in the cucumber body to resist salt–alkali stress in the environment [7]. The tolerance of cucumbers to adversity is closely related to the level of GSH. The application of exogenous GSH can maintain the activity of some antioxidant components, enhance the antioxidant defense system, directly eliminate part of reactive oxygen species (ROS), and thus enhance the tolerance of plants to abiotic stress and oxidative stress [8]. For example, *PtGSTF1* enhances the biomass and salt tolerance of transgenic poplar trees by regulating their hydrolytic enzyme activity, modifying cell walls, maintaining homeostasis, and eliminating ROS [9]. The GST gene of quinoa can balance the endogenous hydrogen peroxide induced by salt stress under salt stress conditions, alleviating the damage caused by hydrogen peroxide to quinoa root cells [10]. *HcGR* can effectively regulate the salt tolerance mechanism of kenaf, which responds to salt stress signals and enhances salt stress resistance by upregulating transcription levels in various tissues [11]. In summary, GST can actively respond to salt stress. Previous studies have confirmed the important role of GST in improving salt tolerance in plants such as rice [12], garlic [13], and tomato [14] in vivo. However, there have been no reports on the mechanism of how GST plays a role in cucumber growth and reducing salt–alkali damage.

Based on the results of RNA-seq sequencing, a candidate gene CsGy3G01390.1 was screened from the glutathione pathway. It was named *CsTAU1* due to its GST structural domain. *CsTAU1* could significantly responded to salt–alkali stress, and was upregulated in ‘D1909’ and downregulated in ‘D1604’, Therefore, we speculated that it is related to the salt–alkali resistance of ‘D1909’. However, there have been no reports on the response mechanism of *CsTAU1* under salt–alkali stress. *CsTAU1* was cloned using the cDNA of ‘D1909’ leaf as a template, and the functional identification of *CsTAU1* overexpressing plants and wild-type under salt–alkali stress was conducted to explore the expression of *CsTAU1* under salt–alkali stress, laying a foundation for studying the molecular mechanism of salt–alkali-tolerant cucumber and breeding new varieties of salt–alkali-tolerant cucumber.

## 2. Materials and Methods

### 2.1. Experimental Materials

The cucumber cultivar ‘D1909’ (with salt–alkali-tolerant) and the cucumber cultivar ‘D1604’ (with salt–alkali-sensitive) were used in this experiment, and the seeds were provided by the College of Horticulture and Landscape Architecture of Heilongjiang Bayi Agricultural University (Heilongjiang, China) [15].

### 2.2. Cloning and Sequence Analysis of CsTAU1

We searched for the coding sequence (CDS) sequence of *CsTAU1* in the cucumber genome database, designed primers using Primer 5.0 (Table S1), and performed polymerase chain reaction (PCR) amplification using the ‘D1909’ leaves cDNA as a template, with an annealing temperature of 57.5 °C. The amplification product was detected by 1% agarose gel electrophoresis, and the target bands were cut off under ultraviolet light, and the target bands of *CsTAU1* gene was recovered using the full gold gel recovery kit. The gel recovery product of *CsTAU1* gene was connected to the p EASY-T3 vector, cultured, and turbid bacterial solution was used as a template for PCR testing. The positive bacterial solution that was successfully identified will be sent to Qingke Co., Ltd. (Harbin, China). for sequencing.

Bioinformatics analysis of *CsTAU1* was obtained by national center for biotechnology information (NCBI). A phylogenetic tree was built using Mega 7.0.

### 2.3. Subcellular Localization of CsTAU1

Based on the CDS sequence with the termination codon removed, primers that carry the cleavage sites of *BamHI* and *EcorI* were designed (Table S1). GFP green fluorescent vector was selected as the expression vector, a fusion expression vector 35S-CsTAU1-eGFP was constructed, Trans1-T1 was transformed into *Escherichia coli* and identified. The extraction and transformation of Arabidopsis protoplasts are detailed in Supplementary File S1. They were observed using a laser confocal microscope (TCS SP2 Leica, Berlin, Germany) [16].

### 2.4. Relative Expression of CsTAU1 in Different Tissues of Cucumber

The salt–alkali soil in the Daqing area (45°46′ N, 124°19′ E) is sulfate soda salt–alkali soil. This study simulated the sulfate soda salt–alkali soil under greenhouse conditions, and prepared a mixed salt–alkali solution in the following proportions: NaCl, Na_2_CO_3_, NaHCO_3_, and Na_2_SO_4_ at a molar ratio of 1:1:9:9. Then, the total salt concentration was calculated to be 80 mmol·L^−1^ based on the Na+ content, and the pH value of the solution was adjusted to 8.9 [17].

Plastic pots with a diameter of 30 cm and a height of 40 cm were selected. Each bucket contained 20 kg of sterile soil (purchased from the Saltusheng Binghe Material Distribution Office in Daqing City, China) and 10 plants of 1 variety. The sowing depth of cucumber is 2 cm, and the sowing conditions are 25 °C and 16 h during the day, 18 °C and 8 h at night, with a relative humidity of 70% in the air. When the cucumbers were at the three-leaf stage, they were sprayed with the configured saline–alkali solution, and with distilled water as the control group. They were sprayed once every 3 days, until the liquid on the surface of the leaves began to drip. This was repeated a total of 3 times. Then, the samples were taken at 1, 6, 12, 24, 48, and 72 h after treatment, frozen in liquid nitrogen, and stored at −80 °C for the determination of gene expression. There were three replicates.

Online tool Primer3plus was used for primer design; the primers used are shown in Table S1, with *CsEF1* as the reference gene α (Gen Bank Access Number: XM:004138916) [18]. The experiment had a total of 3 biological replicates, with a relative expression level of 2^−ΔΔCT^, calculated using the relative quantitative analysis method [19], and Student’s *t* test was used for sample comparison. “*” represents significant difference between the treatment group and control, and “**” represents extremely significant difference between the treatment group and control.

### 2.5. CsTAU1 Expression under Different External Factors

When the seedlings had grown to three true-leaf seedlings, different stress treatments and hormone induction treatments were carried out. Abscisic acid (ABA)-induced treatment: foliar spraying with 100 µmol/L ABA [20]; Salicylic acid (SA)-induced treatment: foliar spraying with 0.5 mmol/L SA; Methyl jasmonate (JA)-induced treatment: foliar spraying with 100 µmol/L JA; cold treatment (low temperature stress): the day and night temperatures were set to 10 ± 0.5 °C/5 ± 0.5 °C with a 16 h photoperiod, the light intensity was 4000 lx, and the low temperature stress was 10 days; Drought stress (PEG): each plant was irrigated with 50 mL of 40% PEG4000, and leaves were harvested 8 days after treatment; Disease treatment (Cor): inoculated Corynespora multicaulis on cucumber leaves by spray method, and the inoculation concentration was 1 × 10^5^ conidia/mL; And leaves were harvested 24 h after treatment. For each of the above treatments, 5 plants with consistent growth were selected as replicates, and leaves were taken as experimental materials after 24 h treatment. After rapid freezing with liquid nitrogen, the samples were stored at −80 °C for analysis of *CsTAU1* gene expression patterns induced by external factors. The expression level detection method is the same as that in Section 2.4.

### 2.6. Genetic Transformation of Cucumber by CsTAU1

Using the full-length cloned primers (Table S1), CsTAU1 was amplified by PCR using pEASY-T3-CsTAU1 plasmid as a template, and the PCXSN-1250 vector was cleaved using XcmIsingle enzyme [21]. T4 ligase connected the target fragment to the vector and transformed it into *Escherichia coli*. The PCXSN-CsTAU1 overexpression vector was calculated through bacterial liquid PCR and sequencing identification, and the calculated overexpression vector was transferred into *Agrobacterium tumefaciens* LBA4404 using the freeze–thaw method. The genetic transformation technique for cucumber in reference [22] was used with minor modification. The seed coat of ‘D1909’ and ‘D1604’ was peeled of, disinfected with 70% alcohol and sodium hypochlorite, and incubated in germination medium for 48–72 h. After agrobacterium infection, the cotyledon nodes were transferred into co-culture medium, and the co-culture process required 48 h of dark cultivation. Afterwards, to differentiation medium was transferred, and the external environment was adjusted (light cycle of 16 h/8 h, constant temperature cultivation at 26 °C); differentiation cultivation occurred for about 28 days. The composition of differentiation medium is 4.43 g MS, 30 g Sucrose and 2 g Phytagel; 1 L of water was added and the pH was adjusted to 5.8. After sterilization, 4 mL Cephalo sporin, 500 μL ABA, 500 μL 6BA and 200 μL phosphinothricin (PPT) were added. The resistance and concentration of PPT were 100 mg·L^−1^, and cucumber cotyledons were transformed. Robust differentiated buds were selected and transferred into rooting culture medium; after the main root grew, they were domesticated and cultivated. The domesticated, genetically modified seedlings were put into an illuminating incubator and acclimated for 15 days before planting them in a greenhouse. Molecular identification of transgenic plants was carried out using PCR and qRT-PCR techniques.

Overexpression (OE), antisense expression (AS) vectors and an empty vector were constructed for the genetic transformation of ‘D1604’ and ‘D1909’. The ‘D1909’ T0 generation obtained a total of 19 OE series, 7 AS series, and 3 empty vector series. Three OE lines (lines 1, 4, and 11) with the highest expression of *CsTAU1* and three AS lines (lines 1, 2, and 7) with the lowest expression were used for further research. The ‘D1604’ T0 generation obtained a total of 15 OE series, 15 AS series, and 3 empty vector series. The three OE lines (lines 1, 2, and 4) with the highest expression of *CsTAU1* and the AS lines (lines 2, 3, and 4) with the lowest expression were selected. The mixed salt–alkali solution treatment of genetically modified cucumber was the same as above, and the empty vector was used as control. The transgenic leaves were used to determine gene expression levels in the glutathione pathway and physiological indicators. Each treatment had three repetitions.

### 2.7. Measurement of Physiological and Biochemical Indicators

Physiological indicators of overexpressing plants were measured, and the methods for measuring chlorophyll and CAT were based on the experimental guidance compiled by Zhao Shijie [23]. The specific detection methods were slightly modified. The GST detection used the GST assay kit (Solarbio), and the α-naphthylamine content was detected using the test kit (Solarbio), both purchased from Nuo He Ltd (Cheng Du, China).

### 2.8. Statistical Analysis

All data were analyzed using SPSS Statistics (Version 19.0; RRID: SCR_002865), Origin and Microsoft Office Excel 2018 (v16.0.11001.20049). The “*” presents extremely significant, *p* < 0.05; The “**” presents *p* < 0.01.

## 3. Results

### 3.1. Cloning and Bioinformatics Analysis of CsTAU1

**Figure 1:**
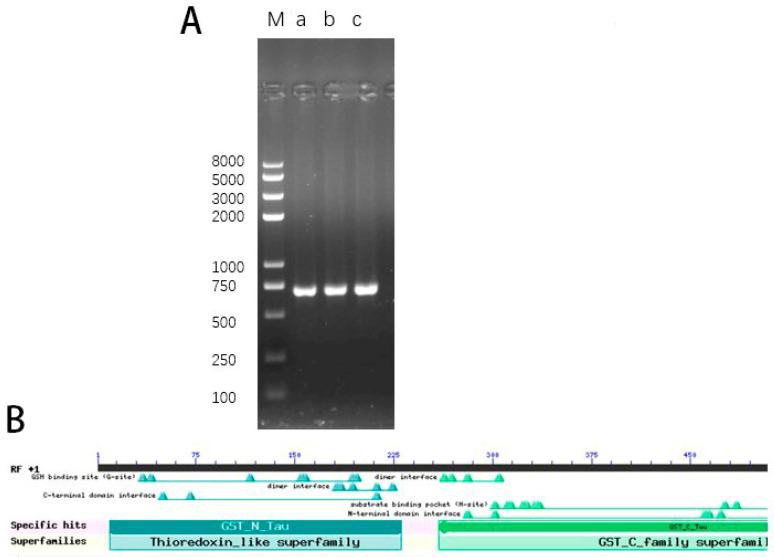
Cloning of *CsTAU1*. (**A**) Gel electrophoresis of *CsTAU1*; (**B**) CsTAU1 protein domain; M: DNA marker 2K; a, b, c: PCR product.

The CDS sequence of candidate gene *CsTAU1* was obtained from the cucurbit genomics database. Using the cDNA of ‘D1909’ as a template, CsTAU1-F and CsTAU1-R were used as primers for PCR amplification, and the purpose strap was obtained (Figure 1A). After purification, it was connected to the cloning vector (pUC18), and the sequencing results showed that it was identical to the CsGy3G011390.1 sequence of the gourd genome database gene with a total length of 675 bp, indicating successful cloning of the *CsTAU1*. The *CsTAU1* protein had one GST-N-Tau domain and one GST-C-Tau domain (Figure 1B), so that it was named *CsTAU1*.

### 3.2. Subcellular Localization of the CsTAU1

**Figure 2:**
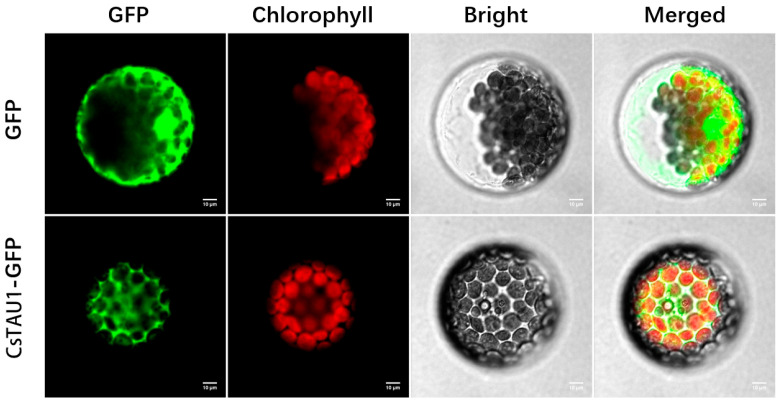
Subcellular localization of CsTAU1 protein. Images show protoplasts prepared from 3- to 4-week-old Arabidopsis leaves expressing CsTAU1-pGII-EGFP (bottom row) or pGII-EGFP (upper row). Bright-field illumination, GFP fluorescence, chlorophyll fluorescence, and an overlay of GFP and chlorophyll fluorescence are shown. Scale bars, 10 μm.

The CsTAU1-GFP fusion expression vector and GFP empty vector were, respectively, transferred into Arabidopsis protoplasts and observed under confocal microscopy. The results showed that CsTAU1-GFP green fluorescence was distributed in the cytoplasm (Figure 2), indicating that CsTAU1 belongs to cytoplasmic proteins.

### 3.3. Phylogenetic Tree of CsTAU1

**Figure 3:**
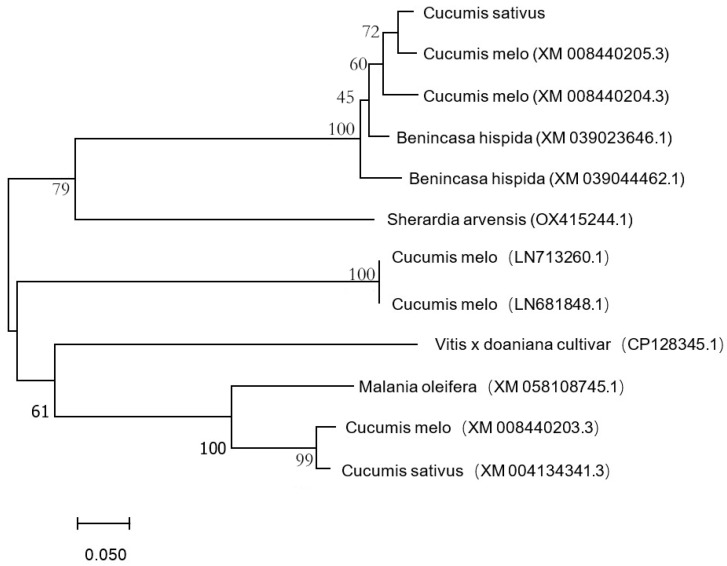
Phylogenetic analysis of *CsTAU1*.

Multiple alignment of *CsTAU1* sequence and construction of phylogenetic tree were performed using the neighbor-joining method with MEGA7.0. As shown in Figure 3, *CsTAU1* has the closest genetic relationship with melon (*Cucumis melo* L. XM 008440205.3).

### 3.4. Relative Expression of CsTAU1 in Different Tissues of Cucumber under Salt–Alkali Stress

**Figure 4:**
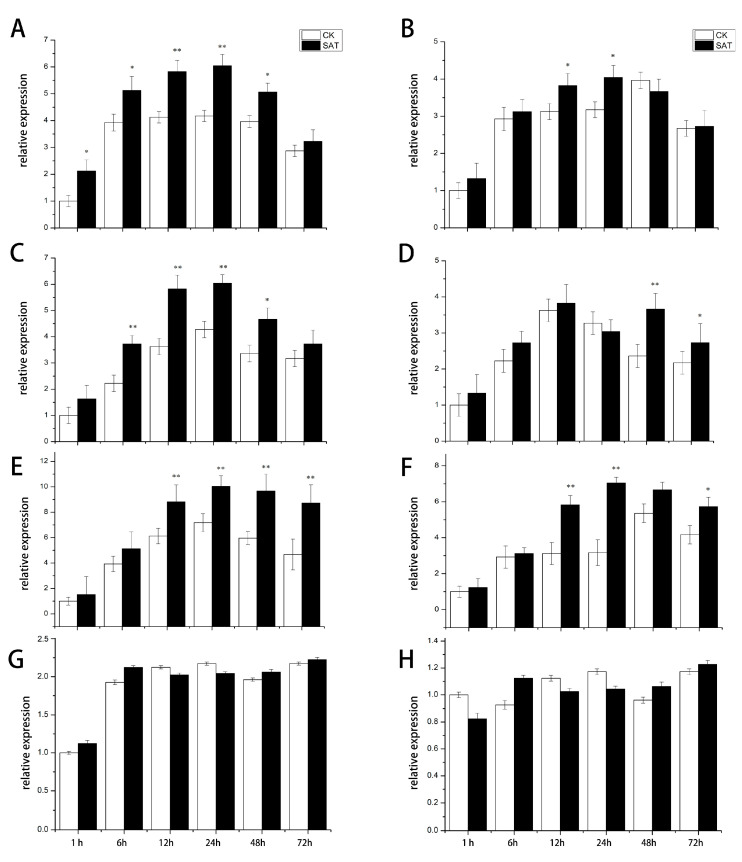
Relative expression of *CsTAU1* under salt–alkali stress. (**A**) The expression level of *CsTAU1* in ‘D1909’ roots; (**B**) the expression level of *CsTAU1* in ‘D1604’ roots; (**C**) the expression level of *CsTAU1* in ‘D1909’ stems; (**D**) the expression level of *CsTAU1* in ‘D1604’ stems; (**E**) the expression level of *CsTAU1* in ‘D1909’ leaves; (**F**) the expression level of *CsTAU1* in ‘D1604’ leaves; (**G**) the expression level of *CsTAU1* in ‘D1909’ fruits; (**H**) the expression level of *CsTAU1* in ‘D1604’ fruits. The “*” presents *p* < 0.05; The “**” presents *p* < 0.01.

The expression level of *CsTAU1* is different between ‘D1909’ and ‘D1604’ under the salt–alkali stress (Figure 4). In the roots of ‘D1909’, the expression level of *CsTAU1* gradually increased over 1–24 h, reaching a maximum value of 6.02 at 24 h, and then decreased. It showed a linear distribution, which was extremely significantly higher than the control at 12 and 24 h—1.43 and 1.40 higher, respectively (Figure 4A). The expression pattern of *CsTAU1* in the roots of ‘D1604’ was the same as that in the roots of ‘D1909’, but the expression was significantly higher than the control only at 12 and 24 h. There was no significant difference compared to the control at other time points, and the expression level of *CsTAU1* in ‘D1604’ was lower than that in ‘D1909’ at all time points (Figure 4B). The expression level of *CsTAU1* in the stems of ‘D1909’ was extremely significantly higher than that of the control at 6, 12, and 24 h, and it was significantly higher than the control at 48 h, at 1.66, 1.59, 1.44 and 1.39 times that of the control, respectively (Figure 4C). In the stems of ‘D1604’, it was only extremely significantly higher than the control at 48 h, and significantly higher than the control at 72 h (Figure 4D). The relative expression of *CsTAU1* in the leaves of ‘D1909’ gradually increased from 1 to 24 h, and then tended to plateau. The expression levels were extremely significantly higher than those of the control at 12–72 h, which were 1.45, 1.44, 1.63, and 1.99 times higher than those of the control, respectively (Figure 4E). Although the expression pattern of *CsTAU1* in the stem of ‘D1604’ is similar to that in ‘D1909’, the expression level at all time points is lower than that in ‘D1909’ (Figure 4F). In the fruits of ‘D1909’ and ‘D1604’, there was no significant difference in the expression level of *CsTAU1* at each time point compared to the control, indicating that *CsTAU1* was not specifically expressed in the fruits (Figure 4G,H).

### 3.5. Analysis of CsTAU1 Expression under Different External Factors

**Figure 5:**
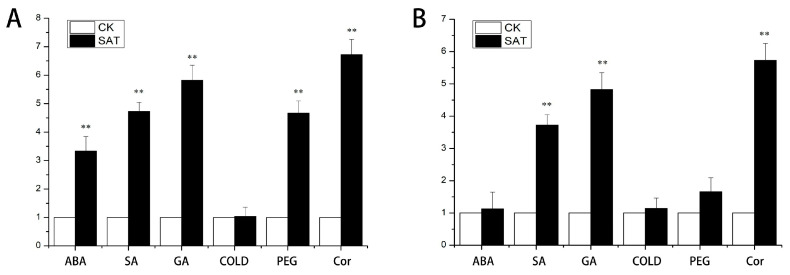
Expression analysis of *CsTAU1* under different hormone and stress conditions. (**A**) Expression analysis of *CsTAU1* in ‘D1909’. (**B**) Expression analysis of *CsTAU1* in ‘D1604’. “**” presents *p* < 0.01; CK is distilled water treatment, SAT is salt–alkali treatment.

The expression of *CsTAU1* under different hormone and environmental stresses was analyzed by qRT-PCR results, as shown in Figure 5. After ABA treatment, *CsTAU1* was specifically upregulated in ‘D1909’, which was extremely significantly higher than the control, 3.36 times higher; the expression level in ‘D1604’ was not significantly different from the control group. After SA and GA treatment, the expression pattern of *CsTAU1* was the same in ‘D1909’ and ‘D1604’, both showing extremely significant upregulation. However, the expression level of *CsTAU1* in ‘D1909’ was higher than that in ‘D1604’; *CsTAU1* is not sensitive to low temperatures and showed no significant difference compared to the control in both ‘D1909’ and ‘D1604’. Under drought stress, *CsTAU1* was extremely significantly upregulated in ‘D1909’, which was 4.55 times higher than the control. However, the expression level in ‘D1604’ was not significantly different from the control, and was 1.53 times higher. The expression levels of *CsTAU1* were similar in both varieties under the stress of Cor, and both showed extremely significant upregulation—6.56 and 6.39 times higher, respectively.

### 3.6. Obtaining CsTAU1 Overexpressed Resistant Plants

**Figure 6:**
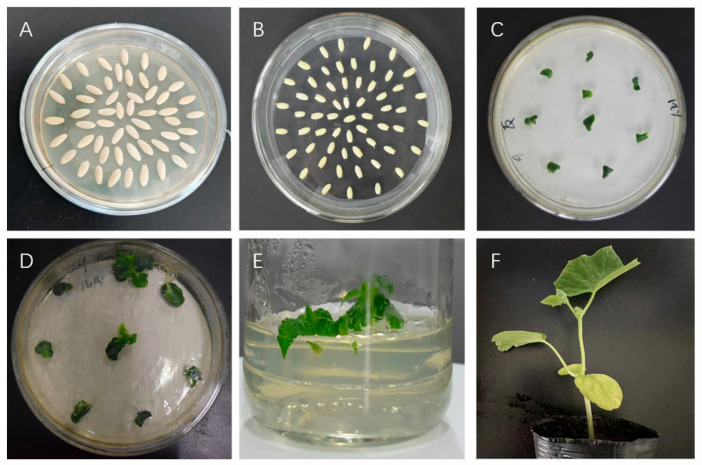
Genetic transformation of overexpression cucumber; (**A**): germination culture of overexpressing cucumber; (**B**): cotyledon node of overexpressing cucumber; (**C**): overexpressing cucumber in differentiation culture; (**D**): differentiated buds grow from overexpressing cucumber; (**E**): rooting culture of overexpressing cucumber; (**F**): overexpressing cucumber domestication culture.

Using the cotyledon nodes of ‘D1909’ and ‘D1604’ as explants, the CsTAU1 was introduced into them through the Agrobacterium infection method. T0 generation transgenic cucumbers were obtained. After domestication, genetically modified seedlings were transplanted into greenhouses (Figure 6).

**Figure 7:**
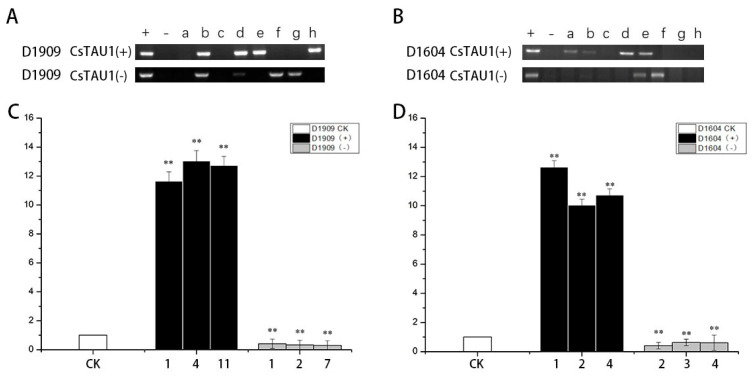
Molecular biological verification of overexpressing cucumbers. (**A**) PCR detection of ‘D1909’ CsTAU1 (+) and ‘D1909’ CsTAU1 (−)-overexpressing plants; (**B**) PCR detection of ‘D1604’ CsTAU1 (+)- and ‘D1909’ CsTAU1 (−)-overexpressing plants; a, b, c, d, e, f, g, h: PCR product. (**C**) qRT-PCR detection of ‘D1909’ CsTAU1 (+)- and ‘D1909’ CsTAU1 (−)-overexpressing plants; (**D**) qRT-PCR detection of ‘D1604’ CsTAU1 (+)- and ‘D1604’ CsTAU1 (−)-overexpressing plants. “**” presents *p* < 0.01.

PCR detection was performed on the obtained ‘D1909’ CsTAU1 (+)-, ‘D1909’ CsTAU1 (−)-, ‘D1604’ CsTAU1 (+)-, and ‘D1604’ CsTAU1 (−)-overexpressing cucumbers. The results are shown in Figure 7. In the (+) lane using the CsTAU1 plasmid as a template, all four overexpressing plants showed bands, while in the (−) lane using water as a template, none of the four overexpressing plants showed bands, and there were clear bands at approximately 700 positions in the detected positive and antisense transgenic plants and positive controls (Figure 7A,B), which were consistent with the target gene fragment, while the negative controls did not have any target bands; this indicates that *CsTAU1* transgenic positive cucumbers were successfully obtained through genetic transformation.

Afterwards, we also tested the expression level of *CsTAU1* in overexpressing cucumbers; they all showed extremely significant differences compared to the wild-type. ‘D1909’ CsTAU1 (+) overexpression in plants (OE1, OE4,OE11) was 12.47 times as high as that in the wild-type; ‘D1909’ CsTAU1 (−) overexpression in plants (AS1, AS2, AS7) was 0.43 times as high as that in the wild-type (Figure 7C); ‘D1604’ CsTAU1 (+) overexpression in plants (OE1, OE2, OE4) was 11.55 times as high as that in the wild-type; and ‘D1604’ CsTAU1 (−) overexpression in plants(AS2, AS3, AS4) was 0.52 times as high as that in the wild-type (Figure 7D).

### 3.7. Performance of CsTAU1 Overexpressing Plants under Salt–Alkali Stress

**Figure 8:**
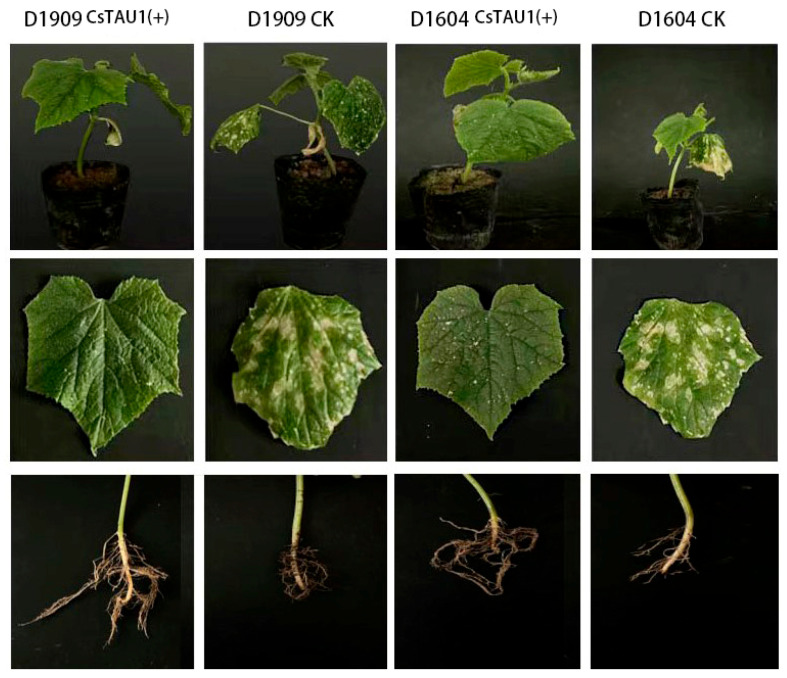
CsTAU1 (+)-overexpression cucumbers growth status under salt–alkali stress.

The growth of ‘D1909’ CsTAU1 (+)- and ‘D1604’ CsTAU1 (+)-overexpressing cucumbers during the three-leaf-stage was identified in salt–alkali stress, while the wild-type was treated with distilled water. Salt–alkali stress leads to stunted plants, wrinkled and yellowed leaves, a short main root system, and few fibrous roots. Compared with wild-type, CsTAU1 (+)-overexpressing plants showed a better overall growth status, and the introduction of CsTAU1 (+) effectively improved the cucumber’s salt–alkali tolerance (Figure 8).

### 3.8. CsTAU1 Reduces the Metabolic Pathway of Salt–Alkali Stress

**Figure 9:**
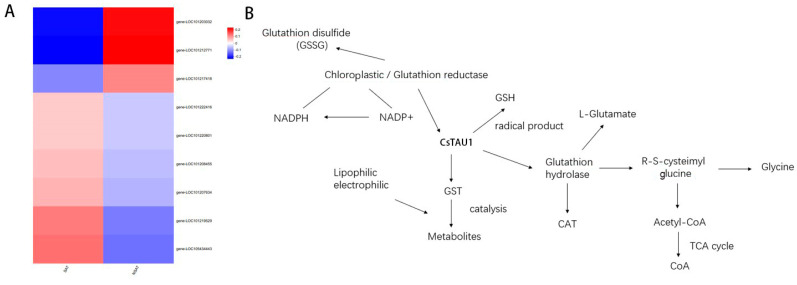
Metabolic pattern diagram of *CsTAU1* in reducing salt–alkali stress. (**A**) Heatmap of SAT and NSAT; (**B**) metabolic pathway diagram.

Based on the functional analysis of *CsTAU1* in KEGG (Figure 9A), a metabolic pattern diagram of *CsTAU1* in reducing salt–alkali stress was drawn (Figure 9B). The upstream gene of *CsTAU1* can promote the conversion of NADP+ to NADPH and eliminate harmful reactive oxygen species in cucumber. Downstream genes stimulate their own immune function by outputting CoA, reducing the damage of salt–alkali stress to cucumbers. This suggests that *CsTAU1* may be a key gene responsible for salt–alkali tolerance in ‘D1909’.

### 3.9. Expression of Key Genes in the Glutathione Pathway in Overexpressing Plants

**Figure 10:**
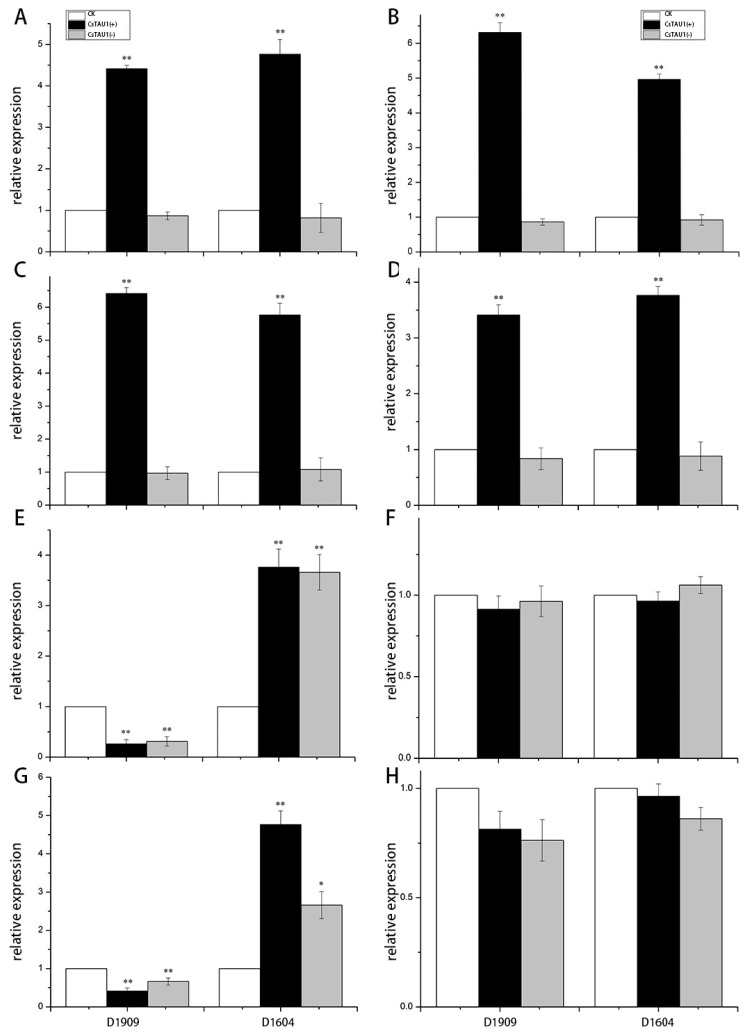
The expression of key genes in the glutathione pathway in *CsTAU1*-overexpressing cucumbers. (**A**) Expression of LOC101219529 under salt−alkali stress. (**B**) Expression of LOC101219529 under non−salt–alkali stress. (**C**) Expression of LOC105434443 under salt−alkali stress. (**D**) Expression of LOC105434443 under non−salt−alkali stress. (**E**) Expression of LOC101203032 under salt−alkali stress. (**F**) Expression of LOC101203032 under non−salt−alkali stress. (**G**) Expression of LOC101212771 under salt−alkali stress. (**H**) Expression of LOC101212771 under non−salt−alkali stress; “*” presents *p* < 0.05; “**” presents *p* < 0.01.

There were nine differentially expressed genes in the glutathione pathway. Among them, LOC101219529 and LOC105434443, which were upregulated in ‘D1909’ and downregulated in ‘D1604’, as well as LOC101203032 and LOC101212771, which were upregulated in ‘D1604’ and downregulated in ‘D1909’, were selected for qRT-PCR analysis to explore whether they have a synergistic effect with *CsTAU1*. Under both salt–alkali and non-salt–alkali conditions, LOC101219529 and LOC105434443 showed extremely significant upregulation in CsTAU1 (+)-overexpressing plants, while their expression levels in CsTAU1 (−)-overexpressing plants were not significantly different from the control. The relative expression of LOC101203032 and LOC101212771 was extremely significantly downregulated in both ‘D1909’ CsTAU1 (+)- and CsTAU1 (−)-overexpressing plants in salt–alkali stress, while it was significantly or extremely significantly upregulated in ‘D1604’ CsTAU1 (+)- and CsTAU1 (−)-overexpressing plants. Under non-salt–alkaline conditions, LOC101203032 and LOC101212771 had similar expression patterns and levels to the control in ‘D1909’- and ‘D1604’-overexpressing plants (Figure 10). It can be inferred that LOC101219529 and LOC105434443 have a certain synergistic effect with *CsTAU1*. Overexpression of *CsTAU1* affects the gene expression levels of LOC101219529 and LOC105434443, but has no effect on the expression levels of LOC101203032 and LOC101212771.

### 3.10. The Effect of Salt–Alkali Stress on Physiological Indicators of CsTAU1-Overexpressing Cucumbers

**Figure 11:**
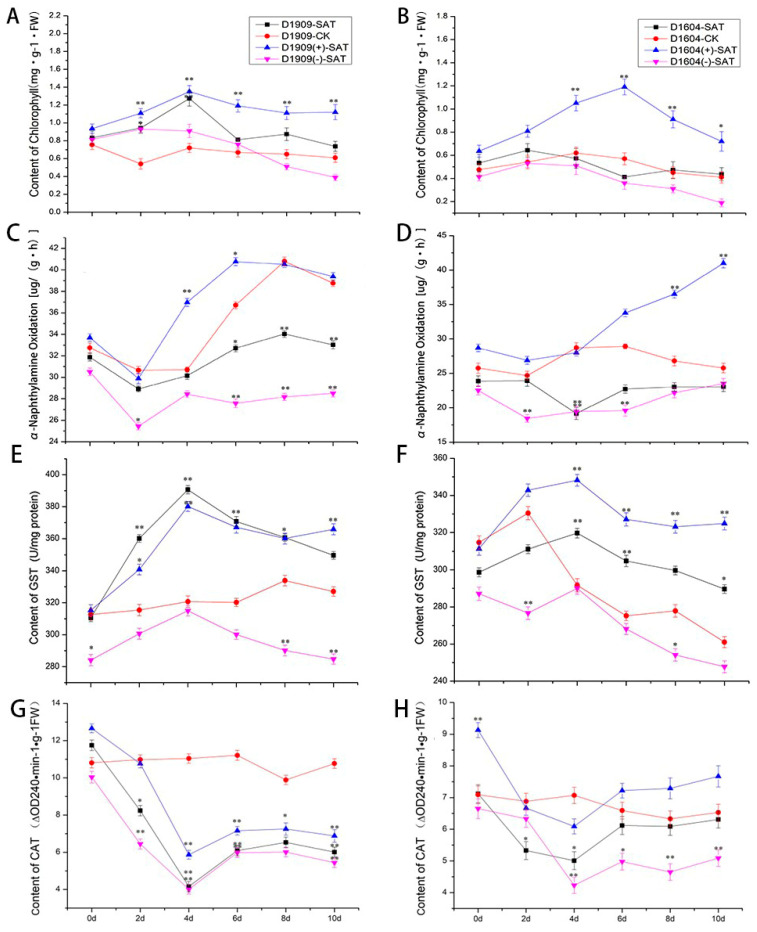
Physiological and biochemical indexes under salt−alkali treatment. (**A**) Chlorophyll content of ‘D1909’. (**B**) chlorophyll content of ‘D1604’. (**C**) α-Naphthylamine oxidation of ‘D1909’. (**D**) α-Naphthylamine oxidation of ‘D1604’. (**E**) GST content of ‘D1909’. (**F**) GST content of ‘D1604’. (**G**) CAT content of ‘D1909’. (**H**) CAT content of ‘D1604’. “*” presents *p* < 0.05; “**” presents *p* < 0.01.

Chlorophyll is a type of plant pigment closely related to photosynthesis, and changes in chlorophyll content under stress can serve as an important indicator of leaf physiological activity. It could be seen that the chlorophyll content of the ‘D1909’ CsTAU1 (+)-overexpressing plants was significantly higher than that in the ‘D1604’ CsTAU1 (+)-overexpressing plants at every point in time. On the 6th day of salt–alkali stress, both reached their maximum values. At this time, the chlorophyll content of the ‘D1909’ CsTAU1 (+)-overexpressing plants was 0.17 mg·g^−1^·FW higher than that of the ‘D1604’ CsTAU1 (+)-overexpressing plants (Figure 11A,B). Transferring an overexpression gene can increase chlorophyll content, while transferring an antisense gene can reduce chlorophyll content. The CsTAU1 protein has been proven to eliminate apoptotic cells in the body and affects root vitality. The α-Naphthylamine oxidation content of ‘D1909’ and ‘D1604’ CsTAU1 (+)-overexpressing plants reached its maximum on the 10th day. The α-Naphthylamine oxidation content showed significant differences in two genotypes of cucumber; it directly affects the oxidative capacity of cucumber roots, and α-Naphthylamine oxidation content is one of the factors that affects the different salt–alkali tolerance levels of two cucumber varieties (Figure 11C,D). GST exerts detoxification and antioxidant functions in cucumber, thereby improving its salt–alkali tolerance. The GST content of ‘D1909’ CsTAU1 (+)-overexpressing plants was higher than that in the wild-type and the water-treated at every point in time in salt–alkali stress. The GST content of ‘D1909’ CsTAU1 (−)-overexpressing plants was lower than that in the wild-type at every point in time except for the 4th day, and was lower than the water-treated at other times (Figure 11E,F). The GST in the two genotypes of cucumber showed different trends of change, indicating that the amount of GST content may be related to the degree of salt–alkali tolerance of the cucumbers. CAT could scavenge H_2_O_2_ and maintain membrane stability, so it is an important enzyme. The transfer of CsTAU1 (+) increased the CAT content in cucumber; this is reflected in the difference in CAT content between ‘D1909’ CsTAU1 (+)- and ‘D1909’ CsTAU1 (−)-overexpressing cucumbers. And on the 4th day, there was the largest difference in CAT content between ‘D1604’ CsTAU1 (+)- and ‘D1604’ CsTAU1 (−)-overexpressing plants, which was 1.80-fold higher. At most time points, the CAT content of ‘D1604’ CsTAU1 (−)-overexpressing cucumbers was lower than that of the wild-type with salt–alkali stress. The trend of CAT changes in ‘D1909’ and ‘D1604’ CsTAU1 (+)-overexpressing plants was similar, but the CAT content in ‘D1909’ was higher than that in ‘D1604’. Therefore, it is believed that the level of CAT content may be related to the cucumber’s ability to withstand salt–alkali stress (Figure 11G,H).

## 4. Discussion

This study screened the gene *CsTAU1*; the expression patterns of *CsTAU1* are different in the two varieties, with significant differences in salt–alkali resistance, upregulated in ‘D1909’ (salt–alkali tolerant) and downregulated in ‘D1604’ (salt–alkali sensitive). It was located in the cytoplasm, which is the main site of plant metabolism and contains some proteins related to redox and free radical neutralization. It is speculated that *CsTAU1* may have the effect of enhancing cucumber antioxidant capacity or scavenging ROS, enhancing cucumber resistance to salt–alkali stress. *CsTAU1* has the closest genetic relationship with melon Cucumis melo (XM 008440205.3). The protein is highly conserved among different species and has high similarity in plants of the same family and genus, indicating that members of the GST family have high conservation during evolution [24].

The transcriptome analysis clearly indicates that there were significant differences in the expression patterns of *CsTAU1* between two varieties with different levels of salt–alkali sensitivity. Under salt–alkali stress, six time points were selected for qRT-PCR detection on different parts of cucumbers, and the expression level of *CsTAU1* in ‘D1909’ was significantly higher than that in ‘D1604’. The leaves were used as the spraying site, and *CsTAU1* showed the most significantly different expression level in leaves. The salt–alkali solution dripping from the leaf edge falls into the soil, and travels through the stems as the transportation site, resulting in significant or extremely significant differences in *CsTAU1* in the roots and stems compared to wild-type. The differential expression of *CsTAU1* was not significant in the fruits of ‘D1909’ and ‘D1604’; this may be because the fruit did not come into direct contact with the salt–alkali solution. The above results reflected the sensitivity of *CsTAU1* in salt–alkali environments, indicating that it is a positive regulatory gene for cucumber salt–alkali tolerance. The differential genes under salt stress were mainly expressed in the leaves, similar to the expression pattern of *CsTAU1* in this experiment [25]. The tissue-specific expression results of the salt–alkali-responsive gene GhVP in cotton showed that the relative expression was highest in leaves and lower in flowers and fibers with salt–alkali [26]. The relative expression level of *CsTAU1* is highest in leaves. It can be preliminarily believed that *CsTAU1* participates in antioxidant and other life activities, and it is a key gene for improving cucumber salt–alkali tolerance.

ABA is a stress hormone in plants that can promote their adaptation to adverse conditions such as drought, salinity, and low temperatures in response to stress signals [27]. ABA processing caused *CsTAU1* to be extremely significantly upregulated in ‘D1909’ compared with wild-type, but there was no significant difference compared with wild-type in ‘D1604’. ABA receptors were different in different cultivars of cucumber. Appropriate amounts of SA have significant effects on cucumber disease, drought, cold, and salt–alkali resistance, which has a significant effect on scavenging excessive free radicals in cucumber leaves. JA can be absorbed through the roots and stems of cucumbers, which is beneficial for promoting the synthesis of several proteins, improving cucumber disease resistance and insect resistance enzymes, and inducing secondary growth of cucumbers [28]. The results of this experiment indicate that compared to ‘D1604’, ‘D1909’ is more sensitive to the induction of SA and JA. There may be a certain relationship between ‘D1909’ and its strong salt–alkali tolerance, but further experiments are needed to prove whether hormone regulation can enhance salt–alkali tolerance in cucumbers. In addition to hormone induction, the expression of *CsTAU1* was validated under three different environmental conditions, and only after PEG treatment were there differences in the relative expression level of *CsGSP9* between ‘D1909’ and ‘D1604’. The domestication of cucumbers under a salt–alkali environment will enhance their ability to resist stress and repair, and this may also contribute to their resistance to salt–alkali stress.

*CsTAU1* is a gene in the cucumber glutathione pathway that plays a positive regulatory role, in which *CsTAU1* has strong antioxidant function. It can also catalyze the combination of substances produced by stress reactions with GSH, enhance the resistance to lipid peroxides and the protection of cell membranes, and play a positive role in stabilizing the intracellular environment [29]. The upstream gene of *CsTAU1* was a glutathione reductase that could regulate chloroplast related functions and promote the conversion of NADP+ to NADPH, which was related to the clearance of harmful reactive oxygen species in the body [30]. It plays an important role in increasing metabolic rate, resisting damage caused by stress, and improving cellular antioxidant capacity, and ultimately producing the metabolite GSSG. *CsTAU1* downstream was a glutathione hydrolase-related gene that could regulate CAT content, produce glucose as an energy substance in cucumber, and regulate CoA output through the intermediate product R-S-cysteine glucose. The glyoxylate cycle oxidizes and decomposes it into CoA [31]. CoA has a certain activating effect on autoimmune function. CoA can promote the body’s immune system to respond to stress substances and participate in the synthesis of intracellular resistance substances [32]; it could be inferred that *CsTAU1* could effectively enhance the tolerance to salt–alkali stress.

To verify whether *CsTAU1* has a synergistic effect with other glutathione-related genes, four genes from the glutathione pathway were screened for qRT-PCR validation. LOC101219529 and LOC105434443 are glutathione S-transfers that can participate in many physiological and metabolic processes in cucumber, such as detoxification of metabolites, regulation of redox status, and maintenance of cell stability [33]. Glutathione S-transferase *GmNAC181* in soybeans can promote root nodule formation and improve salt tolerance in soybeans [34]. Some research experiments have shown that the glutathione-related pathway has been proven to play a role in detoxification metabolism in plants such as corn, wheat, and tomato in vivo [35]. LOC101203032 is an L-ascorbic acid peroxidase, which is one of the important antioxidant enzymes in plant reactive oxygen species metabolism. LOC101212771 is a glutathione peroxidase, which can effectively improve animal immunity and maintain body balance. In this study, the expression patterns of LOC101219529 and LOC105434443 were consistent with *CsTAU1* under salt–alkali stress. It is speculated that overexpression of *CsTAU1* upregulates the expression levels of LOC101219529 and LOC105434443, promoting detoxification and metabolism of secondary products produced by salt–alkali stress in cucumbers, enhancing their antioxidant capacity, and thus increasing their resistance to salt–alkali stress. The relative expression levels between LOC101203032, LOC101212771 and *CsTAU1* are different, indicating that their expression is not affected by *CsTAU1*, and their response to salt–alkali stress is also an independent mechanism of action.

Under salt–alkali stress, cucumber leaves shrink and turn yellow, and chlorophyll content decreases. In this experiment, the transfer of CsTAU1 (+) increased the chlorophyll content in cucumber leaves to a certain extent. Chlorophyll has a strong antioxidant effect, and can eliminate free radicals, reduce oxidative damage caused by salt–alkali stress, and enhance the plant’s ability to adapt to adversity [36]. The oxidative stress of plant roots directly affects the progress of various oxidative reactions in the roots, and also has a certain impact on the absorption and transformation ability of aboveground nutrients [37]. Between two genotypes of cucumber, α-Naphthylamine oxidation showed significant differences, and transferring to CsTAU1 (+) can improve the cucumber root system. α-Naphthylamine oxidation enhances the oxidative capacity of the root system, ensuring the absorption and transportation of nutrients by cucumbers under salt–alkali stress. Cucumbers can metabolize external stress substances, in which GST plays an important role. There are also studies indicating that the content of GST in cucumbers significantly increases after being subjected to stress. This indicates that GST can be induced by salt–alkali expression, protecting cucumbers from damage. This study showed that there were differences in GST content between ‘D1909’ and ‘D1604’, and this may affect the salt–alkali resistance of different genotypes of cucumbers. CsTAU1(+) overexpression can lead to an increase in GST content in cucumbers, while CsTAU1(−) overexpression shows the opposite trend. Therefore, it is speculated that the introduction of CsTAU1 (+) can increase GST content and exercise antioxidant function. This result corresponds to the expression pattern of *CsTAU1* in transcriptome sequencing. The function of CAT is well known; it can scavenge excessive free radicals and have protective and detoxifying effects on plants [38]. It was shown that the transfer of CsTAU1 (+) increased the CAT content, while the CAT content was significantly lower when CsTAU1 (−) was transferred compared to the corresponding wild-type. This indicates that *CsTAU1* has a certain impact on CAT content and improving cucumber’s resistance to salt–alkali stress by reducing H_2_O_2_ to H_2_O. This result is similar to the research results of Nakul and Pal [39,40].

## 5. Conclusions

This study successfully cloned *CsTAU1*. In order to clarify its function, sequence analysis was performed, and fluorescence quantification and physiological and biochemical index analysis were performed on transgenic T_0_ cucumbers. *CsTAU1* has a total length of 675bp and encodes 225 amino acids. It is subcellular and located in the cytoplasm and is closest to Cucumis melo (XM 008440205.3). Compared with ‘D1604’, *CsTAU1* has a special response to SA and PEG in ‘D1909’. The expression patterns of *CsTAU1* are similar to those of genes LOC101219529 and LOC105434443, which are also involved in the glutathione pathway. Transferring CsTAU1 (+) can increase the content of chlorophyll, α-Naphthylamine oxidation, GST, and CAT, enhancing the ability of cucumber to resist salt–alkali stress. This experiment lays the foundation for further exploring the interaction analysis of key genes in the cucumber glutathione pathway under salt–alkali stress and the molecular mechanism of *CsTAU1* in reducing salt–alkali stress.

## Data Availability

The data presented in this study are available on request from the corresponding author.

## Supplementary Materials

The following supporting information can be downloaded at: https://www.mdpi.com/article/10.3390/genes15050613/s1, Table S1: Cloning and qRT−PCR primers; File S1: Extraction and transformation of Arabidopsis protoplasts.
