# Supplementary material for: Cloning and Function Analysis of the CsTAU1 in Response to Salt–Alkali Stress
Source: Genes (Basel). 2024 May 11;15(5):613. doi: 10.3390/genes15050613 (PMC11120846; doi:10.3390/genes15050613)
Supplement: Supplementary file 1 [file genes-15-00613-s001.zip › Supplementary file 1.pdf]

Preparation of reagents related to the extraction of Arabidopsis protoplasts

Cellulase hydrolysate（10 mL，PH=5.7）

Cellulase R10	0.1 g
Mecerozyme R10	0.025 g
Mannitol（0.8 M）	5 mL
KCl（1 M）	0.2 mL
MES（100 mM）	2 mL

W5 solution（100 mL，PH=5.7）

NaCl（5M）	3.08 mL
$CaCl_2$（1 M）	12.5 mL
KCl（1 M）	0.5 mL
Glucose（10 mM）	50 mL
MES（100mM）	2 mL
ddH_2O	31.92 mL

MMg solution（10 mL，PH=5.7）

Mannitol（0.8 M）	5 mL
MgCl2（1 M）	0.15 mL
MES（100mM）	0.4 mL
ddH_2O	4.45 mL

40%PEG4000 solution（10 mL）

Mannitol（0.8 M）	2.5 mL
PEG4000	4 g
$CaCl_2$（1 M）	1 mL
ddH_2O	3 mL

Extraction of Arabidopsis Protoplasts

（1）Sow Columbia wild-type Arabidopsis in germination medium and grow in a light incubator for 7 days;
（2）Transfer Arabidopsis, which sprouts and grows small roots, into a nutrient bowl to continue growing;
（3）Select Arabidopsis thaliana that has not yet bloomed, cut the healthy leaves into thin strips along the direction of the leaf veins;
（4）Put the thin strip into the prepared enzymatic solution, and use tweezers to assist in sedimentation. Conduct enzymatic reaction under dark conditions at 25 ℃ for 4 hours, and the enzymatic solution turns green;
（5）Filter the above enzymatic hydrolysate using a fine mesh nylon membrane;
（6）Centrifuge 100g for 3 minutes;
（7）Gently discard the supernatant to prevent protoplast cell dissection. Slowly add 10ml of W5 solution, wash off the enzyme solution, and centrifuge 100g for 3 minutes;
（8）Repeat step 4, add 10ml of W5 and ice bath for 30 minutes;
（9）Microscopic observation of Arabidopsis thaliana protoplasts to determine if their morphology is intact, with round cells and gaps between them.

35S-CsTAU1-eGFP Plasmid Transformation of Arabidopsis Protoplasts

（1）Take 1mL of the prepared Arabidopsis protoplast cells and slowly add them to a 2mL imported centrifuge tube (not easily adhered to the wall). Centrifuge 100 g for 3 minutes;
（2）Gently discard the supernatant and try to remove it as thoroughly as possible, but do not damage the Arabidopsis protoplast cells;
（3）add 200 μL Resuspend cells in MMG solution, paying attention to cell integrity;
（4）Join 20 μL high concentration plasmid, gently mix well, let stand at 25 ℃ for 5 minutes
（5）Add 220 μL 40% PEG4000, mix gently and let stand for 15 minutes;
（6）Add 1mL of pre cooled W5 solution, slowly flip it up and down, mix well, centrifuge 100g for 1-2 minutes, and gently discard the supernatant;
（7）Repeat step 6;
（8）Add 500 μL mix the pre cooled W5 solution gently and place it flat in a petri dish. Incubate under 25 ℃ light conditions for more than 16 hours to fully express CsTAU1;
（9） Using laser confocal microscopy (Leica, Germany) to observe the expression of fusion proteins.
